# Field emission enhancement of Au-Si nano-particle-decorated silicon nanowires

**DOI:** 10.1186/1556-276X-6-176

**Published:** 2011-02-25

**Authors:** Fei Zhao, Guo-an Cheng, Rui-ting Zheng, Dan-dan Zhao, Shao-long Wu, Jian-hua Deng

**Affiliations:** 1 Key Laboratory of Beam Technology and Material Modification of Ministry of Education, College of Nuclear Science and Technology, Beijing Normal University, Beijing 100875, P. R. China

## Abstract

Au-Si nano-particle-decorated silicon nanowire arrays have been fabricated by Au film deposition on silicon nanowire array substrates and then post-thermal annealing under hydrogen atmosphere. Field emission measurements illustrated that the turn-on fields of the non-annealed Au-coated SiNWs were 6.02 to 7.51 V/μm, higher than that of the as-grown silicon nanowires, which is about 5.01 V/μm. Meanwhile, after being annealed above 650°C, Au-Si nano-particles were synthesized on the top surface of the silicon nanowire arrays and the one-dimensional Au-Si nano-particle-decorated SiNWs had a much lower turn-on field, 1.95 V/μm. The results demonstrated that annealed composite silicon nanowire array-based electron field emitters may have great advantages over many other emitters.

## Introduction

Silicon is one of the most promising candidates and plays a significant role in the micro-electronic field. One-dimensional silicon nanowires (SiNWs) have been fabricated by many approaches, such as chemical vapor deposition [1], laser ablation [2], thermal evaporation [3], chemical etching [4,5] methods, etc., since they were first fabricated via the vapor-liquid-solid (VLS) mechanism [6]. Among the above mentioned methods, for dealing with the challenges that the nanowires should grow aligned in the same direction with high purity, chemical etching method is a simple and convenient way to fabricate pure well-aligned SiNWs.

Fabrication of electron-emitting nano-materials [7-9] and application of electron field emitters on the flat panel displays [10] have attracted much attention on the studies of one-dimensional materials because of their advantages of high aspect ratio, stable structure, and high electron field emission (FE) properties. In the studied field emitters [11-16], SiNW-based emitters [17-21] have been widely studied. In order to improve the FE property, various kinds of modification have been done on SiNWs, such as H_2 _plasma surface treatment of Si nanowires [22,23], Mo-modified Si field emitter [24], Ni-implanted Si samples [25], and IrO_2 _coated on silicon nanotips [26]. Gold is a metal with low resistivity and high structure stability. However, the influence of Au coating and post-annealing treatment on FE properties of silicon nano-structures has not been reported. In this article, the influence of Au coating and post-annealing treatment on FE properties of SiNWs and the enhancement of FE property by modifying as-grown SiNWs to Au nano-particles-decorated SiNWs have been investigated.

### Experimental details

A simple chemical approach was utilized here to synthesize SiNWs [5]. In this procedure, n-type 〈100〉 silicon wafer was used as substrate and ultrasonically cleaned in acetone and ethanol for 5 to 10 min followed by washing in de-ionized water. The cleaned substrates were immersed in AgNO_3_/HF solution to deposit Ag catalyst for 1 min, where the concentrations of AgNO_3 _and HF were 0.01 mol/l and 8%, respectively. Afterward, they were quickly transferred into H_2_O_2_/HF solution to fabricate SiNWs at room temperature, where the concentrations of H_2_O_2 _and HF were 0.6 and 8%, respectively. After 1-h chemical etching, the color of silicon surface became dark, which indicated the formation of SiNWs. The as-grown SiNWs were post-treated in 1% diluted HF solution to remove the SiO_2 _layers coated outside. Au film deposition was carried out by using DC magnetron sputtering technology in Ar atmosphere (1 Pa), and the sputtering current was 20 mA. The thicknesses of the Au films were nominally defined as 20, 60, and 80 nm for different deposition times. Further experiments were done by annealing the SiNWs with Au films at 500, 650, and 800°C, respectively, in a quartz tube furnace and H_2 _atmosphere for 2 h.

The morphology and structure characterization of the nanowires were done by scanning electron microscope (SEM) (S-4800, Hitachi) and transmission electron microscope (TEM) (FEI TECNAI F30, Philips). FE measurements were carried out in the high vacuum chamber with a base pressure of 3 × 10^-7 ^Pa. The distance between cathode and anode was indicated by an electronic digital display indicator and could be adjusted in a scale of 0 to 5 cm. Applied voltages up to 10 kV could be impressed on the flat anode to ensure that the emission current could be tested and detected with a digital multimeter.

## Result and discussion

**Figure 1:**
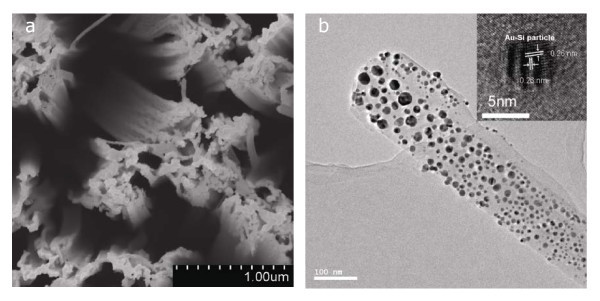
**Microstructures of 20 nm Au film-coated SiNWs**. **(a) **SEM image of as-coated SiNWs, in which Au layer covered on the tip of SiNWs equally; **(b) **TEM Images of 20-nm Au film-coated SiNWs post-annealed at 650°C, inset in **(b) **is the HRTEM image of Au-Si nano-particle. TEM and HRTEM images illustrate that the Au-Si phase had been formed after post-annealing at 650°C and Au-Si nano-particle-decorated SiNWs had been fabricated.

As reported previously [5,21], the as-grown SiNWs are about 10 μm in length and 120 nm in diameter with single crystal structure. In order to be unconfused, SiNWs coated with Au film thicknesses of 20, 60, and 80 nm were labeled as Au20/SiNWs, Au60/SiNWs, and Au80/SiNWs, respectively. Figure 1a shows the SEM image of the top surface of the Au20/SiNWs, from which we could observe that Au nano-film with an average particle size of 51.0 nm has been covered on the top of SiNWs arrays. With the film thickness increasing from 20 to 80 nm, the average size of Au particles changes from 51.0 to 103.6 nm for Au60/SiNWs and 144.5 nm for Au80/SiNWs.

Figure 1b shows TEM image of the 650°C post-annealed Au20/SiNWs. From Figure 1b, it can be seen that Au20/SiNWs are about 120 nm in diameter, and there are many nano-sized particles with the size ranging from 10 to 48 nm being dispersively distributed along the axis of the post-annealed Au20/SiNW. The inset of HRTEM image in Figure 1b illustrates that nano-sized particles on the surface of the post-annealed Au/SiNWs are nano-crystalline particles. The interplanar spacing calculated from HRTEM image of the nanoparticle is approximately 0.26 nm, which can be attributed to the [660] planes of Au_2_Si phase [27]. Compared with as-grown SiNWs [19], the morphology of the post-annealed Au20/SiNW has not been changed much and still shows the wire-shape in which many Au nano-particles covered on the surface of nanowire. These observations indicate that Au-Si nano-particle decorated SiNWs can be fabricated during annealing at a high temperature.

**Figure 2:**
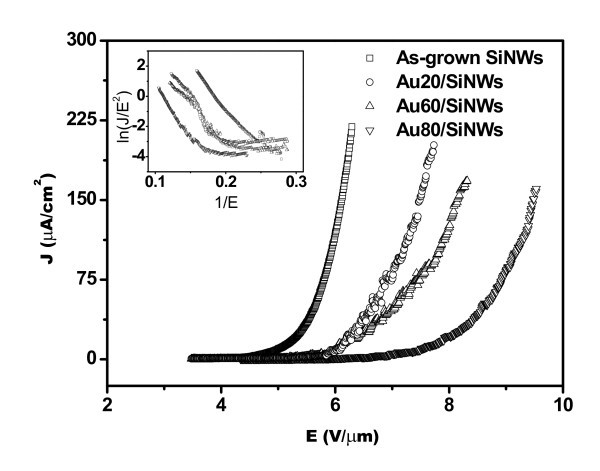
***J*-*E *curves of Au/SiNWs with different thickness, in which thicknesses increase of Au film induces the *J*-*E *curves shifting to higher applied field and makes FE properties worsen**. The inset is corresponding *F*-*N *plots.

The FE properties of SiNWs were measured at the room temperature. The curves in Figure 2 display the emission current density (*J*) of SiNWs and Au/SiNWs as a function of the applied field (*E*), and the inset is *F*-*N *plots of samples. The obtained *J*-*E *curves gradually shift to the highly applied field with the increase of Au film thickness, and turn-on fields (*E*_on_) (which are defined as the field when *J *reaches 10 μA/cm^2^) are 5.01, 6.02, 6.03, and 7.51 V/μm for the as-grown SiNWs, Au20/SiNWs, Au60/SiNWs, and Au80/SiNWs, respectively. The shifting of *J*-*E *curve to the highly applied field and the high value of *E*_on _demonstrate that electrons are harder to emit from the tips of Au nano-particles than that from the tips of SiNWs, and FE properties of SiNWs have been strongly affected because of the deposition of Au film. The tendency can also be observed at higher *J*. When *J *reaches 100 μA/cm^2^, the applied field is 5.93 V/μm for as-grown SiNWs, and increases to 7.20, 7.81, and 9.18 V/μm for Au20/SiNWs, Au60/SiNWs, and Au80/SiNWs, respectively (see Table 1). These results clearly demonstrated that Au film coated to SiNWs makes FE characteristics worsen.

**Table 1:** FE parameters of Au film-coated SiNWs with different thicknesses

Samples	*E*_on_, (V/μm)	*E*_*J = 100*μA/cm^2 ^_(V/μm)	*Φ *(eV)
As-grown SiNWs	5.01	5.93	4.15
Au20/SiNWs	6.02	7.20	4.29
Au60/SiNWs	6.03	7.81	4.51
Au80/SiNWs	7.51	9.18	4.98
Au	-	-	5.55

According to the *F*-*N *theory [28] which is explored to indicate the mechanism of FE, *J *varies exponentially with *E *and work function (*Φ*) of emitting material. With modification, FN Equation 1 can be used to describe the linear relationship between ln(*J*/*E*^2^) and 1/*E*, i.e.,

(1) ln⁡(JE2)=−Bϕ32β−1E+ln⁡(Aϕ−1β2)

where *β *is the field enhancement factor, *A *and *B *are constants equal to 1.54 × 10^-6 ^A eV V^-2 ^and 6.83 × 10^3 ^eV^-3/2 ^V μm^-1^, respectively. Thus, *β *or *Φ *could be calculated via the slope of the straight line of ln(*J*/*E*^2^) and 1/*E*. Because the area of SiNW array is large and the morphology is relatively uniform, little change of the morphology has been made after Au deposition. The authors have the evidence to assume that the value of *β *is approximately equal to each other, and so *Φ *will change for different emitters. According to the *F*-*N *plots given in the inset of Figure 2, *Φ *can be calculated to be 4.15, 4.29, 4.51, and 4.98 eV for as-grown SiNWs, Au20/SiNWs, Au60/SiNWs, and Au80/SiNWs, respectively, as shown in Table 1. FE property of the emitter is highly dependent on their composition, tip sharpness, aspect ratio, conductivity, and work function. High *Φ *makes electron FE difficult and reduces FE property of the emitter. These observations further confirm that Au deposition without annealing is not effective in the improvement of FE property.

**Figure 3:**
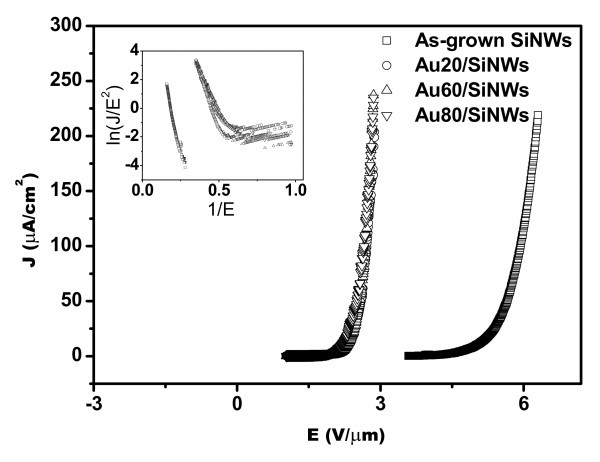
***J*-*E *curves of SiNWs coated with different thicknesses of Au film and post-annealed at 650°C, which show that the 650°C post-annealing processing of Au/SiNWs makes *J*-*E *curves shifting to lower applied field and enhances FE properties of SiNWs**. The similar results have been obtained in Au/SiNWs with different thicknesses. The inset shows corresponding *F*-*N *plots.

**Figure 4:**
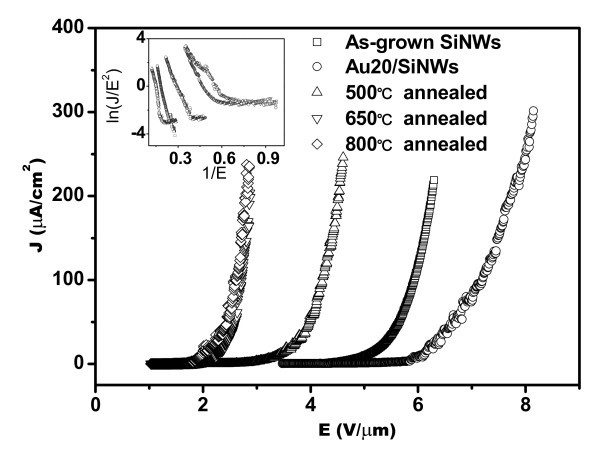
***J*-*E *curves of 20-nm Au film-coated SiNWs post-annealed at different temperatures, which show that the excellent FE properties of Au20/SiNWs with low *E*_on _values have been obtained after post-annealing processing above 650°C**. The inset shows corresponding *F*-*N *plots.

Further examination was carried out via annealing the Au/SiNWs with different thicknesses at 650°C. Figure 3 shows the FE properties of the post-annealed Au/SiNWs with different thicknesses at 650°C. From Figure 3, it can be seen that *J*-*E *curves of the post-annealed Au/SiNWs are overlapping with each other and located at a lower applied field than that of as-grown SiNWs. The corresponding values of *E*_on _are 2.25, 2.31, and 2.19 V/μm for the annealed Au20/SiNWs, Au60/SiNWs, and Au80/SiNWs, respectively, where relative changes of *E*_on _values are very small. At the same time, the FE properties of the post-annealed Au20/SiNWs at different temperatures are depicted in Figure 4 and Table 2, which show that the post-annealing temperature increase makes the *J*-*E *curves of samples move to lower applied field. The similar *J*-*E *curves can be obtained after post-annealing above 650°C. According to the *J*-*E *curves, *E*_on _values of Au20/SiNWs post-annealed at 500, 650, and 800°C are 3.37, 2.25, and 1.95 V/μm, respectively, and the applied fields, at which *J *is 200 μA/cm^2^, are 4.53, 2.88, and 2.79 V/μm, respectively. These results indicate that electrons can emit easily from the tips of the post-annealed Au/SiNWs at a lower applied field, and suggest that the FE properties of Au/SiNWs are remarkably improved due to post-annealing processing.

**Table 2:** FE parameters of Au20/SiNWs before and after post-annealing at different temperatures

Samples	*E*_on _(V/μm)	*E*_*J = 200 *μA/cm^2 ^_(V/μm)
As-grown SiNWs	5.01	6.25
Au20/SiNWs	6.02	7.73
Au20/SiNWs 500°C	3.37	4.53
Au20/SiNWs 650°C	2.25	2.88
Au20/SiNWs 800°C	1.95	2.79

The FE properties of emitters are related with their composition, tip sharpness, aspect ratio, conductivity, and work function. Au has a high work function (*Φ*), i.e. 5.55 eV [29], which is larger than that of Si (about 4.15 eV [30]). When continuous Au layer covered on SiNWs, electron FE comes from the Au tip covered on SiNWs and not from the tip of SiNWs. High work function of the emitter makes electron emission difficult and reduces more the FE property of the emitter. Therefore, *E*_on _values of the Au/SiNWs are higher than that of the as-grown SiNWs, and the FE properties of the Au/SiNWs are worse than that of as-grown SiNWs. However, the post-annealing processing above 650°C for the Au/SiNWs makes the *J*-*E *curves move to lower applied field, and lower *E*_on _values, which is in the range from 1.95 to 2.35 eV, can be obtained. Those prove that the FE properties of Au/SiNWs can be remarkably enhanced by the post-annealing processing above 650°C. TEM and HRTEM images in Figure 1 illustrate that Au-Si nano-particles had been formed at the surface of Au20/SiNWs after post-annealing at 650°C, and that the Au-Si nano-particle-decorated SiNWs had been fabricated. Previous studies show that gold silicide, such as Au_2_Si [31,32] and Au_m_Si_n _[33], have been observed, and some kind of composition has good optical, excellent electron transportation, and FE properties. For the Au_2_Si nano-particle-decorated SiNWs, electrons transport near and on the surface of the composite region. Tunneling effect happens when the energy states are distributed in the band gap and the electrons in the conduction band of the SiNWs can be tunneled [34]. This will make the surface potential barrier height of the emitter to be reduced. On the other hand, the similar enhancement results, in which the differences are very small, have been observed in SiNWs coated by Au film with different thicknesses and then post-annealed at 650°C. It is due to the formation of similar micro-structures of the Au-Si nano-particle-decorated SiNWs. Thus, we have the evidence to believe that uniform Au-Si nano-particle-decorated SiNWs could improve the FE properties of SiNWs by enhancing electron transportation and reducing surface potential barrier height of emitter.

## Conclusions

Au film coating on the tip of SiNWs reduces the FE properties of SiNWs because of Au having high work function. Well-aligned Au-Si nano-particle-decorated SiNW arrays have been fabricated by Au film deposition and post-annealing above 650°C, which have excellent FE properties. The lowest *E*_on _value of the Au-Si nano-particle-decorated SiNWs is about 1.95 V/μm, and *J *can reach 200 μA/cm^2 ^at the applied field of 2.79 V/μm. Improvement of the FE properties may be due to Au-Si nano-particle decoration on the top surface of SiNWs, which enhances electron transportation in the SiNWs and reduces the surface potential barrier height of the emitter. These results indicate that the post-annealed Au/SiNW arrays would be used in the field of flat panel displays in the future.

## Abbreviations

FE: field emission; SEM: scanning electron microscope; SiNWs: silicon nanowires; TEM: transmission electron microscope; VLS: vapor-liquid-solid.

## Competing interests

The authors declare that they have no competing interests.

## Authors' contributions

FZ carried out the studies of sample fabrication, acquisition of data, analysis and interpretation of data, and drafted the manuscript. GAC carried out the conceiving of the study, revised the manuscript and given final approval of the version to be published. RTZ participated in the analysis and interpretation of data and revised the manuscript. DDZ, and SLW participated in the sample fabrication and acquisition of data. JHD participated in the acquisition of field emission data. All authors read and approved the final manuscript.
